# The science of communication, the art of medicine

**Published:** 2016-03-31

**Authors:** Marcel D’Eon

**Affiliations:** University of Saskatchewan, Saskatoon, SK

We have all heard people talk (or write) about the art and science of medicine. Sometimes we mean that there’s a certain art or craft to clinical medicine, the application of science to real people in real situations. Sometimes we are referring to the unpredictable human side of medicine, the relationships and rapport building essential to clinical and professional practice. Whether we refer to clinical judgment or communication skills, what we imply is that in science we “know” but in art we are “winging it.” Definitions of science include words such as facts, principles, laws, truth, knowledge, and systematic, while definitions of art include creative skill, imagination, appreciation, and beauty. In Roze des Ordons et al. (this issue, 2016) we read that a misplaced and inappropriate word might leave a more lasting and painful scar than a surgeon’s sloppy scalpel, or as they put it, “… unhelpful communication can cause iatrogenic suffering, with a lasting impact upon patients and families and residual uncertainty and emotional distress amongst trainees, [thus] difficult discussions should be considered *as seriously as an invasive procedure.*” Our interactions with patients can have lasting and profound consequences so we should not be “winging it.” We need to move the art and craft of communications to a higher level where principles and laws of human behaviour and complex interactions are systematically learned and skillfully applied.

There is much at stake here. Patient compliance, satisfaction with care, and even the outcomes of treatment^1^ are associated with the communication patterns of physicians. Through several recent personal examples, I was made keenly aware of the importance of communication in the workplace for all professionals. One such example involved a student who casually reported an unflattering observation of a group of students which turned out to be untrue. We needed to have a frank but tense conversation about making inferences and then talking about them as if they were accurate perceptions of reality. In another example, having discovered a major scheduling issue, I asked my colleague to request that the speaker with whom he was communicating present on a different day. He was clearly unenthusiastic and expressed his reluctance and embarrassment to impose upon our guest. Nevertheless, I politely but firmly insisted he make the request knowing I was asking him to do something he would find very difficult (with really no other good option). In yet another situation, a friend encountered a colleague of his who has been disruptive in several committees causing dysfunction and great discontent among other committee members. Since no one has yet spoken directly with this person about his behaviour, I encouraged my friend to consider being the one to address this with him. His deep sigh and facial expressions told me plenty. In situations such as these, both clinical and professional, given what is at stake, is it good enough that we are satisfied that we just “wing it?”

Interpersonal and communication skills, be they with patients, other health professionals, colleagues, or community agencies, can be learned, re-learned, and perfected. We must not let our personal ignorance of the field of communication science blind us to the vast amounts of rigorous and systematic knowledge to be found in these disciplines. While we currently have endocrinologists, physiologists, and all sorts of highly trained and educated medical and biological specialists in our medical schools, rarely are experts in communication being employed to teach and assess communication and interpersonal skills. Maybe it’s time that we did.

In the book, *Difficult Conversations: How to Discuss What Matters Most*,^2^ I’ve learned about assumptions we often make (with disastrous consequences), how to reframe difficult conversations in more positive ways, about perspective taking and how our beliefs manage and shape our perceptions (and not the other way around), and especially about inviting others to tell their stories while we listen. Reflecting on the lessons in the book, I learned again the lessons of Aesop’s fable about the north wind and the sun, how gentle persuasion can often succeed where force and determination fail.^3^ And of course I learned some practical and effective approaches to difficult conversations. Now I am much more knowledgeable but painfully aware of how I should have spoken or behaved differently in a challenging situation (ignorance is bliss sometimes), as I am not yet skilled enough to apply all of my knowledge at the fast pace of real life.

For our students and residents and, yes, even for our faculty, to transform the art of communication into the science of communication we need to acknowledge that there is much to learn and that there are experts with this sophisticated knowledge who are willing and able to teach. We need to believe that it is valuable use of student and faculty time to learn to be expert communicators. And we need to allocate sufficient time to do it right. Otherwise we will most certainly continue to “wing it.”

In this issue we have another broad array of articles. Roze des Ordons et al. (2016) wrote about palliative and end of life (EOL) communication in postgraduate medicine. Using a survey and focus groups with trainees as well as interviews with clinical faculty and medical educators, they found that trainees were least confident and least satisfied with their instruction about the emotional impact of emergencies and discussing organ donation. Direct observation with feedback, small group discussion, and viewing videos of personal consultations were perceived as effective yet infrequently identified as instructional methods. The narrative data reported uncertainty, anxiety, feelings of abandonment, and moral distress amongst trainees. Their study echoes previous research calling for more and better education in palliative and EOL communication.

Kidd et al. (2016) described an interdisciplinary group workshop designed around a discomfiting oil portrait intended to trigger provocative conversations among health care students and practitioners about vulnerable patients. They argued that difficult conversations among professionals about affective responses to vulnerable persons are possible in a collaborative context using well-chosen works of visual art. Perhaps this approach would be well suited to aspects of communication training for palliative and end of life care.

Koszycki et al. (2016) evaluated the feasibility and benefits of an 8-week peer-led mindfulness meditation program (MMP). Though compliance was suboptimal, the MMP decreased levels of stress and enhanced mindfulness, self-compassion and altruism from baseline to post-study. Implementation at other sites may be a challenge as it remains to be seen how much emphasis medical schools will place on the mental health and general well-being of their students.

Lindsay et al. (2016) reported on differences between physicians who do and those who do not frequently participate in continuing professional development (CPD). Not surprisingly, non-attenders indicated less satisfaction with present opportunities and requested development in newer approaches. The authors concluded that while there are high levels of satisfaction with current CPD, a substantial number of physicians wanted new options such as personal study and on-line resources. It remains to be seen if new approaches to CPD will change non-attenders to participants.

Roy et al., working at the University of Manitoba, investigated whether the pre-medical Grade Point Average (GPA), Medical College Admission Test (MCAT), internal examinations and National Board of Medical Examiners (NBME) scores were correlated with and predict the Medical Council of Canada Qualifying Examination Part I (MCCQE-1) scores. Analyzing data from almost 400 students, they found that the MCCQE-1 had a moderate-to-large positive correlation with NBME scores and internal examination scores but a low correlation with GPA and MCAT scores. Stepwise regression analysis showed that 59.2% of the variation in the MCCQE-1 was accounted for by the NBME, but only negligible variation came from the GPA and the MCAT.

Dagnone et al. (2016) explored the feasibility and validity of high-fidelity simulation in competency-based assessment in postgraduate medical education. They were able to demonstrate a successful pilot of a multi-centre, 3-station simulation-based OSCE for the assessment of resuscitation competence in post-graduate Emergency Medicine trainees.

Boutis et al. (2016), using Rasch Measurement Theory, compared the interpretation difficulty of normal versus abnormal radiographs of a set of common pediatric radiographs and were also able to identify case features that were associated with item difficulty. While abnormal images were in fact more difficult to interpret, normal images were not uniformly easy. They concluded that including a sizable proportion of normal cases may be of benefit to learners.

Steinmetz et al. (2016) investigated the extent and the characteristics of bedside ultrasound teaching in medical schools across Canada. Many medical schools have integrated bedside ultrasound teaching in their undergraduate curriculum. The majority of vice-deans responding supported the integration of bedside ultrasound education into the medical school curriculum but sited numerous barriers.

Lougheed (2016) asked and then answered this provocative question: “Is this clinical exam (MCCQE Part II) truly protecting Canadian patients by assuring them that ‘that their doctors, wherever they are in Canada and whatever their medical specialty, meet the same demanding, consistent standards,’^4^ or is it an outdated requirement, a historical artifact?” You may be able to answer this question but you may enjoy reading the response from the Medical Council of Canada even more.
